# Diversity Arrays Technology (DArT) and next-generation sequencing combined: genome-wide, high throughput, highly informative genotyping for molecular breeding of *Eucalyptus*

**DOI:** 10.1186/1753-6561-5-S7-P54

**Published:** 2011-09-13

**Authors:** Carolina Sansaloni, Cesar Petroli, Damian Jaccoud, Jason Carling, Frank Detering, Dario Grattapaglia, Andrzej Kilian

**Affiliations:** 1 EMBRAPA Genetic Resources and Biotechnology - EPqB Final W5 Norte 70770-917 Brazilia DF and Dep. Cell Biology, Universidade de Brazilia – UnB, Brazil; 2 Diversity Arrays Technology Pty Ltd - PO Box 7141, Yarralumla, ACT 2600, Australia; 3 EMBRAPA Genetic Resources and Biotechnology – Estação Parque Biológico, 70770-910, Brazilia, DF and Genomic Sciences Program - Universidade Católica de Brasília - Brazilia, Brazil

## Background

Wider genome coverage and higher throughput genotyping methods have become increasingly important to meet the resolution and speed necessary for a variety of applications in genomics and molecular breeding of forest trees. Developed more than 10 years ago [1], the Diversity Arrays Technology (DArT) has experienced an increasing interest worldwide for it has efficiently satisfied the requirements of throughput, genome coverage and inter-specific transferability for over 40 different plant species to date, including *Eucalyptus*[2] and recently *Pinus* (Dione Alves-Freitas, this meeting). DArT is based on genome complexity reduction using restriction enzymes, followed by hybridization to microarrays to simultaneously assay hundreds to thousands of markers across a genome. Genome complexity reduction for genotyping has now been taken to another level when combined to next generation sequencing (NGS) technologies. Such a strategy has been used for rapid SNP discovery in different organisms [3], and proposed as a way to genotype with RAD (Restriction-associated DNA) sequencing [4]and recently by a similar method generally termed GbS (Genotyping-by-Sequencing)[5]. In this work we assessed the power of the now well established DArT marker platform in combination with Illumina short read sequencing to generate a linkage map for a segregating outcrossed F1 population derived from *E. grandis* BRASUZ1, the donor of the *Eucalyptus* reference genome.

## Methods

A segregating population of 89 individuals derived from the intra-specific cross BRASUZ1 x M4D31 was provided by Suzano company. Correct parentage of all individuals was certified by microsatellite genotyping. DNA samples of parents and progeny were processed for the conventional array-based DArT genotyping as described earlier [2]to generate marker data for comparative analysis with the NGS based DArT data. For the sequencing based DArT genotyping two complexity reduction methods optimized for several other plant species at DArT PL were used: PstI_ad/TaqI/HpaII_ad and PstI_ad/TaqI/HhaI_ad with TaqI restriction enzyme used to eliminate a subset of PstI -HpaII and PstI-HhaI fragments, respectively. PstI-site specific adapter was tagged with 92 different barcodes enabling encoding a plate of DNA samples to run within a single lane on an Illumina GAIIx. PstI adapter included also a sequencing primer, so that the tags generated were always reading into the genomic fragments from the PstI sites. After the sequencing run the FASTQ files (full reads of 77 bp) were quality filtered using the threshold of 90% confidence for at least 50% of the bases and in addition filtered more stringently for barcode sequences. The filtered data were split into their respective target (individual) data using barcode splitting script. After producing various QC statistics and trimming of the barcode the sequences were aligned against the reference *Eucalyptus grandis* genome available in Phytozome. The output files from alignment (generated using Bowtie software) were processed using an analytical pipeline developed by DArT PL to produce "DArT score" tables and "SNP" tables. A linkage maps was constructed with JoinMap 3.0 [6]using the microarray-based DArT markers, DArT NGS markers, and 40 microsatellites of known map position as anchors. A parallel analysis exclusively meant to estimate the total number of potential SNPs within the short read tags was carried out using CLC genomic workbench v4.6 software [7]with a minimum read coverage of 6 and minimum variant frequency of 25%.

## Results

The microarray-based DArT platform yielded 1,088 high quality markers of which 505 (46.4%) segregated in a 1:1 pseudo-testcross while the remaining 583 (53.6%) segregated 3:1. This relatively lower number of markers when compared to other Eucalyptus mapping populations was expected. Not only it is an intra-specific cross but also involves BRASUZ1, a know self (S1) individual with a lower level of sequence heterozygosity. DArT genotyping using NGS technology yielded 2,835 polymorphic presence/absence markers, almost three-fold the number produced by the microarray platform. Of these, 2,449 markers mapped to the 11 chromosome scaffolds with an average of 222 markers per scaffold, while the remaining 386 markers fall out of the 11 scaffolds, potentially allowing the localization of a fraction of the still unassembled smaller genome scaffolds. In total, an integrated linkage map with 564 DArT markers, 1,930 DArT-NGS and 29 microsatellites was preliminarily built. Furthermore, from the 148 million reads generated (~10.5 Gb), 83.6 million (6.1 Gb) were successfully mapped on the Eucalyptus reference genome. Although a very large number of SNPs can be identified when all reads combined are mapped, only a fraction that displays sufficient coverage allows robust scoring at the individual level. Still, over 1,500 SNPs could be confidently genotyped providing a further advantage of adding co-dominant markers to the already large number of dominant markers obtained.

## Conclusion

These initial results show that the combined use of DArT as a robust genome complexity reduction method with optimized barcoded NG sequencing protocol provides at least three fold more dominant markers than the conventional microarray-based DArT method and an additional set of co-dominant SNPs. We are now genotyping a much larger set of distantly related individuals of a training population to be used for Genomic Selection (GS). The possibility of delivering large numbers of both dominant and co-dominant markers with the same platform will enable fitting dominance effect in predictive models therefore increasing the selection accuracy.
